# Circulating granulysin levels in healthcare workers and latent tuberculosis infection estimated using interferon-gamma release assays

**DOI:** 10.1186/s12879-016-1911-6

**Published:** 2016-10-18

**Authors:** Pham Huu Thuong, Do Bang Tam, Shinsaku Sakurada, Nguyen Thi Le Hang, Minako Hijikata, Le Thi Hong, Phan Thi Minh Ngoc, Pham Thu Anh, Vu Cao Cuong, Ikumi Matsushita, Luu Thi Lien, Naoto Keicho

**Affiliations:** 1 Hanoi Lung Hospital, Hanoi, Vietnam; 2 Department of Biochemistry, Hematology and Blood Transfusion, Hanoi Lung Hospital, Hanoi, Vietnam; 3 Bureau of International Medical Cooperation, National Center for Global Health and Medicine, Tokyo, Japan; 4 NCGM-BMH Medical Collaboration Center, Hanoi, Vietnam; 5 Department of Pathophysiology and Host Defense, The Research Institute of Tuberculosis JATA, Tokyo, Japan; 6 Department of National Tuberculosis Program, Hanoi Lung Hospital, Hanoi, Vietnam; 7 Hanoi Department of Health, Hanoi, Vietnam; 8 National Center for Global Health and Medicine, Tokyo, Japan

**Keywords:** Granulysin, Serum concentration, Latent tuberculosis infection, Gene expression, Genotype, Biomarker

## Abstract

**Background:**

Granulysin (GNLY) is produced by human lymphocyte subpopulations and exhibits antimicrobial activity against *Mycobacterium tuberculosis*. We examined the association between GNLY levels in blood and latent tuberculosis (TB) infection.

**Methods:**

Latency of TB infection among Vietnamese healthcare workers was estimated using interferon-gamma release assays (IGRA), and serum GNLY concentrations were measured using enzyme-linked immunosorbent assays. The levels of *GNLY* expression in whole blood and the presence of *GNLY* alleles with the exon-4 polymorphism rs11127 were also determined using PCR-based methods.

**Results:**

Among 109 study participants, 41 (37.6 %) were IGRA positive and had significantly lower serum GNLY concentrations compared with IGRA-negative participants (adjusted mean, 95 % confidence interval; 2.03, 1.72–2.44 vs. 2.48, 2.10–2.92 ng/ml, *P* = 0.0127; analysis of covariance). Serum GNLY concentrations and TB antigen-stimulated interferon-gamma values were weakly inversely correlated (*r* = −0.20, *P* = 0.0333). Serum GNLY concentrations varied with *GNLY* genotypes even after adjustment for gender and age (adjusted *P* = 0.0015) and were moderately correlated with *GNLY* expression in blood cells (*r* = 0.40, *P* < 0.0001). In subsequent analyses, low serum GNLY concentrations were significantly associated with IGRA status (adjusted odds ratio and 95 % confidence interval, 0.55 and 0.31–0.98, respectively), although *GNLY* genotype and mRNA levels were not.

**Conclusions:**

Decreased GNLY, presumably at the protein level, is linked to the immunological condition of latent TB infection.

## Background

According to global estimation, latent *Mycobacterium tuberculosis* (MTB) infection is present in approximately one third of the human population (~2 billion people) [1]. Latent infection follows prolonged survival of MTB despite containment by host defense mechanisms [2] and contributes significantly to the risk of overt disease following reactivation of MTB under conditions of compromised immunity [3], which presents a major obstacle to achieving global tuberculosis (TB) control.

Interferon-gamma release assays (IGRA) are diagnostic of TB infection and operate on the principle that MTB-specific antigens provoke immune responses in whole blood after the establishment of infection [4]. IGRA offer more specific detection of latent TB infection (LTBI) than tuberculin skin test in many circumstances [5]. Accordingly, QuantiFERON-TB Gold In-tube enzyme-linked immunosorbent assay (ELISA)-based IGRA are recommended in multiple current guidelines, including those of the United States Center for Disease Control and Prevention for testing LTBI among people who are at risk of TB infection (i.e., healthcare workers) [6].

As an effector molecule, the saposin family protein granulysin (GNLY) is involved in protective immunity, and is released from natural killer (NK) cells, NKT cells, γδ T cells, and cytotoxic T lymphocytes (CTLs). This molecule directly eliminates extracellular MTB and intracellular bacteria in the presence of perforin by causing osmotic shock and inducing apoptosis [7]. Recently Walch et al*.* [8] suggested that GNLY initially penetrates infected cells and delivers bactericidal granzymes to intracytoplasmic bacteria. Hence, in the presence of GNLY, granzymes eliminate bacteria independently of host cell death.

Precursor and mature GNLY proteins of 15 and 9 kDa, respectively, have been identified, and mature GNLY is found with perforin and granzymes in cytotoxic granules. Moreover, a previous report has shown that the 9-kDa mature protein is mainly secreted from activated CTLs, whereas the 15-kDa precursor protein is found in plasma under physiological conditions [9]. Although the precursor protein may have more potent chemotactic and inflammatory activities, its physiological function remains poorly understood [10].

Circulating GNLY levels may be a useful indicator of overall host cellular immunity [11]. Moreover, GNLY expression has been identified as a marker for outcomes in cancer and organ transplantation patients and is an important mediator of damage in skin diseases [10]. In TB, plasma GNLY levels were significantly lower in patients with active disease than in healthy individuals and increased after successful anti-TB treatment [12]. In an animal study, GNLY expression was associated with protection after vaccination against TB in cattle [13]. However, it is not clear whether GNLY levels are associated with human LTBI. In the present study, we investigated the relationship between serum GNLY concentrations and LTBI status as diagnosed using IGRA, determined *GNLY* mRNA expression in blood cells, and analyzed *GNLY* genetic polymorphisms.

## Methods

### Study population and data collection

Healthcare workers were recruited from ten district TB-centers in Hanoi city. These TB centers are responsible for implementing directly observed treatment short-course (DOTS) programs for half of the city’s population under the management of a municipal TB hospital (Hanoi Lung Hospital). Staff members who did not participate in the 2007 LTBI survey in this hospital [14] were also recruited and those who were employed for less than one year were excluded.

All study participants were interviewed and demographic information and factors associated with TB exposure were recorded using a structured questionnaire. Histories of Bacillus Calmette-Guérin (BCG) vaccination were obtained and confirmed according to the presence of BCG scars. Peripheral blood was collected for IGRA and for analyses of serum concentrations, mRNA expression, and polymorphisms of *GNLY*. Participants were encouraged to take chest X-rays and sputum tests, when IGRA positive status was identified. The participants were also given the chances of LTBI treatment after consultation with TB specialists.

### Interferon-gamma release assays

IGRA for TB are used to estimate interferon-gamma induction by MTB-specific antigens (TB antigens). In this study, ELISA-based IGRA were performed using the third version assay QuantiFERON-TB Gold In-Tube™ (Cellestis, Victoria, Australia). Briefly, whole blood was collected in separate tubes that were not pre-coated with any stimulants for the negative control, and pre-coated with mitogen for the positive control or TB antigens. After 18 h incubation at 37 °C, concentrations of interferon-gamma in plasma supernatants were measured using the ELISA method. Interferon-gamma concentrations were calculated by subtracting negative control values from TB antigen-stimulated values, and the cut-off value was 0.35 IU/ml. The testing procedure was carefully monitored [15] and quality control was included in each run according to the manufacturer’s instructions.

### Serum granulysin assays

Both 15- and 9-kDa forms of serum GNLY were measured using a sandwich ELISA system with anti-GNLY (RB1) mouse IgG1κ as the capturing antibody and anti-GNLY-biotin (RC8) mouse IgG1κ as the detecting antibody as described previously [11]. Recombinant human (rh)-GNLY was used as a standard and culture supernatants from *GNLY* (15 kDa)-expressing Cos-7 cells were used as positive controls. GNLY concentrations were calculated from a standard curve of serially diluted rh-GNLY standards. This ELISA system is specific for GNLY and has a detection limit of approximately 20 pg/ml [11].

### Quantitative real-time polymerase chain reaction (PCR) of granulysin mRNA in whole blood

Whole blood samples of 2.5 ml were collected into PAXgene™ Blood RNA tubes (PreAnalytiX, Hombrechtikon, Switzerland) and stored as recommended by the manufacturer. Total RNA was then extracted using PAXgene™ Blood RNA Kits (QIAGEN, Hilden, Germany) and reverse transcribed using random nonamers (TaKaRa, Shiga, Japan) and SuperScript III reverse transcriptase (Invitrogen, Carlsbad, CA, USA). Quantitative PCR was performed using the TaqMan® Gene Expression Assay Hs00246266_m1 for *GNLY* (Applied Biosystems, Foster City, CA, USA) with a CFX96 real-time PCR system (BioRad, Hercules, CA, USA). *GNLY* expression was normalized to that of *GAPDH* using the ΔΔCt method [16] and mRNA expression levels of *GNLY* were expressed relative to control cDNA.

### Single nucleotide polymorphism analysis

Genomic DNA samples were extracted from blood cells using QIAamp DNA Blood Mini Kits (QIAGEN) and were subjected to PCR amplification for analysis of single nucleotide polymorphism (SNP) of the *GNLY*. Among the Kinh Vietnamese people, using the 1000 Genomes Database, twenty-one SNPs were identified in *GNLY* and the 5′ region up to 1000 bp from the transcription start site of the reference mRNA sequence (NM_006433.4) [17]. All SNPs were in strong linkage disequilibrium (D’ = 1, *r* ^*2*^ > 0.85) with each other, except for the SNP rs2043760 in intron 1 (D’ = 1, *r* ^*2*^ = 0.7). A non-synonymous SNP rs11127 (C/T) of the exon 4 (NM_006433.4) was selected and genotyped as a representative SNP. Genomic DNA was amplified using the primers 5′-GGAGGTATCAGTCTAGAG**G**TA-3′ and 5′-GCTAAAGTCCATCTGCTCAA-3′, and a mismatch nucleotide (bold) was introduced in the sense primer to generate a *Kpn* I restriction enzyme site when the rs11127 allele was C. Genotype was determined according to the length of PCR products after digestion with *Kpn* I (TaKaRa) and C and T alleles gave 207- and 229-bp fragments, respectively.

### Statistical analysis

Proportions between two study groups were compared using the chi-squared test. First, the serum GNLY concentrations measured by ELISA were used to check their distribution, and Wilcoxon rank-sum test was used to compare the distribution between groups. Then serum GNLY concentrations and *GNLY* mRNA levels were used for further analyses after logarithmic transformation of the values to approximate normal distribution. Since GNLY levels are influenced by gender and age, measurements of GNLY in the two groups were compared using analysis of covariance (ANCOVA) to adjust for covariates after performing unpaired t-tests. Relationships between GNLY levels and other parameters were assessed using Pearson’s correlation coefficients and logistic regression models were used to assess risk factors for positive IGRA status. Allele-number dependent changes in gene expression were identified using univariate and multivariate linear regression models. Variables with biological meaning and with *P* values < 0.2 in univariate analyses were included in multivariate models. Differences and correlations were considered significant when *P* < 0.05, unless otherwise specified. Statistical analyses were performed using STATA version 11 (StataCorp, College Station, TX, USA).

## Results

### Characteristics of the study population

Among 109 healthcare workers, 53 (46.8 %) were recruited from the 225 staff members working in a municipal hospital that specializes in TB, and 150 of these members participated in the 2007 TB infection survey. This hospital has 260 inpatient beds, receives approximately 5000 inpatients per year, performs 120 consultation per day in the hospital outpatient department, and performs 7000 examinations per year in the community. In addition, 56 (51.4 %) healthcare workers were recruited from TB units that are located in district centers of preventive medicine, which function to implement DOTS at the grass roots level.

All participants answered questionnaires and provided blood samples. No participants showed physical signs of active TB. Almost half of the participants were less than 30 years old and 76.2 % of participants were female. Approximately 10 % of participants were obese with a body mass index (BMI) ≥ 25.0 and 50 % of participants had histories of BCG vaccination, as indicated by the presence of BCG scars. Although 78.0 % of participants declared frequent exposure to TB in their working places, only 42 (38.5 %) wore masks frequently (Table 1).

**Table 1:** Characteristics of the study population (*n* = 109)

	Number	Percent
Age (in years)		
20–29	54	49.5
30–39	26	23.9
40–49	15	13.8
≥ 50	14	12.8
Sex		
Men	26	23.8
Women	83	76.2
Body mass index		
< 18.5	7	6.4
18.5–24.9	92	84.4
≥ 25.0	10	9.2
Education level		
High school	3	2.8
Primary, secondary, pre-university	76	69.7
University and higher	23	21.1
Others	7	6.4
BCG vaccination scar		
No	51	46.8
Yes	54	49.5
NA	4	3.7
Ever diagnosed as TB		
No	107	98.2
Yes	2	1.8
Ever treated for TB		
No	107	98.2
Yes	2	1.8
Years served in healthcare profession		
< 2	26	23.9
2–4.99	37	33.9
5–9.99	21	19.3
≥ 10	25	22.9
Job		
Medical doctor	19	17.4
Nurse	55	50.5
Laboratory Technician	11	10.1
X-ray technician	2	1.8
﻿ Other	22	20.2
Current working place		
Lung hospital	53	48.6
District TB center	56	51.4
Current working area		
Outpatient department	9	8.3
TB ward	57	52.3
Non-TB ward	11	10.1
TB bacteriology laboratory	11	10.1
Non-TB bacteriology laboratory	1	0.9
Non-bacteriology laboratory	2	1.8
Administration	11	10.1
Other	5	4.6
NA	2	1.8
Current TB exposure in working place		
Never	8	7.3
Rare	3	2.8
Occasionally	12	11.0
Frequently	85	78.0
Do not know	1	0.9
Mask use		
Never	11	10.1
Rare	3	2.8
Occasionally	52	47.7
Frequently	42	38.5
NA	1	0.9

*TB* tuberculosis, *NA* not available, *BCG* Bacillus Calmette–Guérin

### Serum granulysin levels and interferon-gamma levels after stimulation

**Fig. 1:**
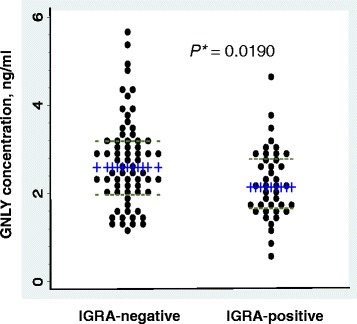
Distribution of serum GNLY concentrations in IGRA-negative and IGRA-positive groups. Blue horizontal lines (+++), median values; green line (---), interquartile range; *by Wilcoxon rank-sum test; GNLY, granulysin; IGRA, Interferon-gamma release assays

**Fig. 2:**
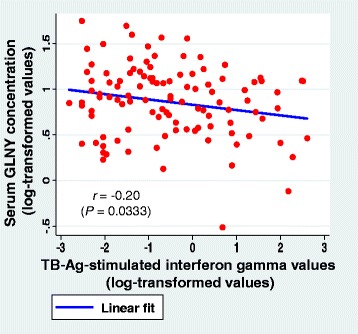
Serum GNLY concentrations and interferon-gamma levels after stimulation with the TB antigens in IGRA. GNLY, granulysin; TB-Ag-stimulated interferon-gamma values, tuberculosis antigen values minus negative-control values in the blood. The scales of x- and y- axes are based on the values after logarithmic transformation of the original units; IU/ml for IGRA values and ng/ml for serum GNLY concentrations

Forty-one individuals were IGRA positive (37.6 %; 28.5–47.4 %). IGRA-positive status did not differ significantly between subjects from the TB hospital and district TB centers (data not shown). Distribution of GNLY levels was significantly different between IGRA-positive and negative groups (median, interquartile range; 2.14, 1.70–2.82 vs. 2.60, 2.01–3.23 ng/ml, *P* = 0.0190 by Wilcoxon rank-sum test) (Fig. 1). After logarithmic transformation of GNLY values and adjustment for age and gender using ANCOVA, GNLY concentrations were significantly lower in the IGRA-positive group than in the IGRA-negative group (adjusted mean, 95 % confidence intervals (95 % CI); 2.03, 1.72–2.44 vs. 2.48, 2.10–2.92 ng/ml; *P* = 0.0127; Table 2). Moreover, GNLY concentrations were negatively correlated with interferon-gamma levels after stimulation with the TB antigens used in IGRA (*r* = −0.20, *P* = 0.0333; Fig. 2).

**Table 2:** GNLY concentrations and IGRA results

	IGRA negative	IGRA positive
	(*n* = 68)	(*n* = 41)
Before adjustment^a^		
Mean value^b^ of GNLY concentrations	2.53	2.10
95 % CI	2.32–2.77	1.84–2.36
*P* value^c^	0.0116	
After adjustment^a^		
Mean value^b^ of GNLY concentrations	2.48	2.03
95 % CI	2.10–2.92	1.72–2.44
*P* value^d^	0.0127	

*GNLY* granulysin, *IGRA* interferon-gamma release assays, *95 % CI* 95 % confidence intervals

^a^adjustment for age and sex

^b^GNLY concentrations with the original unit (ng/ml) are shown after transforming back

^c^unpaired t-test after logarithmic transformation of GNLY concentrations

^d^ANCOVA after logarithmic transformation of GNLY concentrations

The levels of TB exposure assessed by the questionnaire [14] were not different between IGRA-positive and negative groups (data not shown).

### Serum granulysin concentrations, granulysin mRNA expression levels in blood, and polymorphisms of rs11127 SNP in exon 4 of the granulysin gene

**Fig. 3:**
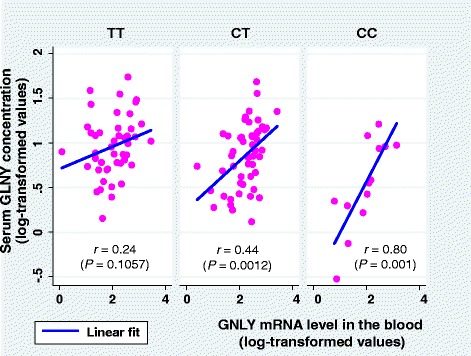
Serum GNLY concentrations and *GNLY* mRNA gene expression levels in the blood by different genotypes of rs11127 SNP of the exon 4 of *GNLY* gene. GNLY, granulysin; mRNA, messenger RNA; SNP: single nucleotide polymorphism. The scales of x- and y- axes are based on the values after logarithmic transformation of the original units; arbitrary unit for *GNLY* mRNA levels, and ng/ml for serum GNLY concentrations

A positive correlation was identified between serum GNLY concentrations and *GNLY* mRNA expression levels in the blood (*r* = 0.40, *P* < 0.0001; table not shown). In the subgroup analysis, serum GNLY concentrations correlated with *GNLY* mRNA, as the number of C allele was increased (*r* = 0.24, *P* = 0.1057 for TT genotype; *r* = 0.44, *P* = 0.0012 for CT; and *r* = 0.80, *P* = 0.001 for CC; Fig. 3). Serum GNLY levels varied significantly with genotype after adjustment for gender and age (*P* = 0.0015) and were low in participants with the CC genotype (adjusted mean, 95 % CI; 1.60, 1.25–2.05 ng/ml), intermediate in participants with the CT genotype (2.23, 1.8–2.64), and high in participants with the TT genotype (2.48, 2.12–2.92). Moreover, numbers of C alleles in these individuals were inversely associated with serum GNLY levels in univariate and multivariate regression models (Table 3). However, no clear differences in mRNA expression were found between participants with CC, CT, and TT genotypes either before or after adjustment for gender and age (adjusted mean, 95 % CI; 9.21, 6.11–13.87; 12.43, 9.30–16.61; and 9.78, 7.46–12.68 arbitrary units, respectively; *P* = 0.0994). Moreover, univariate and multivariate regression models did not show any signification associations between numbers of C alleles and *GNLY* mRNA expression levels (Table 3).

**Table 3:** *GNLY* polymorphisms and other factors associated with serum GNLY concentrations or *GNLY* mRNA expression levels in blood in univariate and multivariate regression models (*n* = 109)

	Univariate			Multivariate		
	Coefficient	95 % CI	*P* value	Coefficient	95 % CI	*P* value
a) Polymorphism of rs11127 SNP in exon 4 of the *GNLY* was inversely associated with serum GNLY concentrations (log-transformed values)						
Age (increased by one year)	0.00	−0.01 to 0.01	0.985	0.00	−0.01 to 0.01	0.666
Gender (female vs. male)	0.04	−0.13 to 0.22	0.628	0.08	−0.09 to 0.25	0.359
Genotype (increased by one C allele)	−0.18	−0.29 to −0.07	0.001	−0.19	−0.30 to −0.08	0.001
b) Polymorphism of rs11127 SNP in exon 4 of the *GNLY* was not associated with *GNLY* mRNA expression levels (log-transformed values) in blood						
Age (increased by one year)	0.00	−0.01 to 0.01	0.870	0.00	−0.01 to 0.01	0.883
Gender (female vs. male)	−0.33	−0.61 to −0.05	0.021	−0.34	−0.62 to −0.06	0.019
Genotype (increased by one C allele)	0.03	−0.15 to 0.21	0.725	0.06	−0.12 to 0.24	0.532

*GNLY* granulysin, *SNP* single nucleotide polymorphism, *95 % CI* 95 % confidence interval

### Factors associated with latent tuberculosis infection estimated using interferon-gamma release assays

In both univariate and multivariate regression models, significant reverse associations were shown between IGRA-positive status and serum GNLY concentrations [unadjusted odds ratio (OR), 95 % CI; 0.55, 0.35–0.89; and adjusted OR, 95 % CI; 0.55, 0.31–0.98], whereas the number of C alleles and *GNLY* mRNA levels were not associated with IGRA-positive status. High BMI was independently associated with IGRA-positive status (Table 4). In addition, GNLY concentrations were significantly lower in IGRA-positive group than in IGRA-negative group, even after the effect of C alleles was considered (ANCOVA; *P* = 0.006, data not shown).

**Table 4:** Serum GNLY concentrations and other factors associated with IGRA-positive status in univariate and multivariate logistic regression models (*n* = 109)

	No (%)	Univariate		Multivariate^a^	
		OR	95 % CI	OR	95 % CI
Age (by year)		1.02	0.98–1.06	1.03	0.98–1.09
Sex					
Male	11/26 (42.3)	Reference		Reference	
Female	30/83 (36.1)	0.77	0.31–1.89	0.83	0.26–2.65
BMI					
18.5–24.9	31/92 (33.7)	Reference		Reference	
< 18.5	3/7 (42.9)	1.48	0.31–7.01	3.99	0.53–29.92
≥ 25.0	7/10 (70.0)	**4.59**	**1.11–18.99**	**8.43**	**1.37–51.75**
Job					
Others	4/22 (18.2)	Reference		Reference	
Doctor	10/19 (52.6)	5.00	1.22–20.46	3.37	0.69–16.52
Nurse	22/55 (40.0)	3.00	0.89–10.06	2.51	0.56–11.18
Technician	5/13 (38.5)	2.81	0.59–13.34	1.48	0.22–10.03
Working place					
Non-TB	8/30 (26.7)	Reference		Reference	
TB	32/77 (41.6)	1.96	0.77–4.94	1.70	0.55–5.22
GNLY concentration (by ng/ml)		**0.55**	**0.35–0.89**	**0.55**	**0.31–0.98**
*GNLY* mRNA expression (by one unit)		0.95	0.88–1.02	0.98	0.88–1.08
Genotype (by number of C allele)		0.84	0.47–1.51	-	-

*GNLY* granulysin, *IGRA* interferon-gamma release assays, *OR* odds ratio, *95 % CI* 95 % confidence interval, *BMI* body mass index, *TB* tuberculosis; significant associations are presented in bold

^a^Multivariate analyses included age, sex, BMI, job category, working place, GNLY concentration, and *GNLY* mRNA expression levels

## Discussion

In the present study, we investigated GNLY in LTBI as identified using IGRA among healthcare workers in Hanoi, Vietnam. Serum GNLY concentrations in the IGRA-positive group were significantly lower than those in the IGRA-negative group. Moreover, serum GNLY concentrations were significantly positively correlated with *GNLY* mRNA expression levels in blood and were dependent on the number of C alleles of the rs11127 SNP in exon 4 of *GNLY*, whereas *GNLY* mRNA expression levels were not dependent on the number of C alleles of the rs11127 SNP in exon 4 of *GNLY*. Of the three parameters (SNP genotype, mRNA expression, and concentrations of GNLY in blood), only circulating levels of GNLY protein were significantly negatively associated with IGRA-positive status.

GNLY, a major antimicrobial molecule, has been shown to preferentially bind and disrupt cholesterol-poor membranes of microbes [10]. However, no homolog has been found in rodents, and investigations using human samples are required to elucidate the significance of GNLY in vivo, as shown in the present study. In a recent study [8], bactericidal mechanisms involving cooperation of GLNY and granzymes have been clarified. Specifically, GLNY delivers granzymes to intracellular bacteria leading to oxidative damage through cleavage of bacterial antioxidants. Thus, GNLY shows both cytolytic and bactericidal activities with other effector molecules such as granzymes and perforin, although GNLY acts independently at micro-molar concentrations and under hypotonic or acidic conditions [18, 19].

Since CTLs play crucial roles in the containment of MTB in TB granulomas [2, 19], GNLY appears to be an important effector molecule in human TB infection, alone and in cooperation with granzymes and perforin. In a previous study in which the same ELISA system was used [11], GNLY concentrations of 244 presumably TB-unexposed healthy Japanese individuals were 3.7 ± 3.2 ng/ml (mean ± standard deviation), and these values were even higher than those in IGRA-negative Vietnamese individuals presumably resistant to TB infection in our study. It suggests that TB exposure may decrease serum GNLY levels, and that LTBI state may further suppress the level by some unknown mechanism, though effects of ethnicity and other background such as comorbidity and nutritional status should be taken into consideration and it is difficult to draw any conclusions from direct comparison between different studies. Moreover, previous studies of active TB disease have shown that GNLY levels in the blood vary with clinical stage [12, 20]. Although serum GNLY concentrations were lower in the LTBI group than in the non-LTBI group, this GNLY concentration (median, interquartile range; 2.144, 1.703–2.824 ng/ml) was higher than that shown previously in newly diagnosed active TB (median ± standard error, 1.511 ± 0.287 ng/ml) and relapsed TB (1.458 ± 0.329 ng/ml) patients in our collaborative study using the same ELISA system [20]. Potential interpretations of low GNLY levels in TB infection are as follows. First, the present data may reflect sequestration of GNLY-producing cells: Lymphocytes with high GNLY expression accumulate at the sites of latent infection. At these sites, continuous cross-talk between the host immune system and the pathogen [21] may facilitate GNLY turnover, and accumulation of these GNLY-producing lymphocytes potentially results in a relative decrease in circulating GNLY in infected individuals. Moreover, it is well known that MTB-specific CTLs accumulate in areas surrounding infected epithelioid cells of TB granulomas [19, 22] and may also occur in individuals with LTBI. This condition may differ from that of chronic TB, which is characterized by persistent inflammation and active clinical manifestations, and is associated with diminished GNLY in granuloma-associated CTLs, resulting in impaired CTL activities [23]. Second, protein levels of GNLY were negatively associated with interferon gamma release in the present study, whereas *GNLY* expression levels and *GNLY* polymorphisms were not, suggesting that GNLY may lack stability in TB infection, and its rates of metabolism or degradation may be affected by chronic inflammation and immune reactions in TB. Finally, during the very early stages of TB infection, NK and NKT cells may destroy pathogens and infected cells [24], and as innate and intermediate immune cells, they express both GNLY and granzymes. Hence, limited expression of GNLY in these cells may lead to failure to eliminate MTB and it may cause IGRA-positive status. However, although frequencies of the C allele of the rs11127 *GNLY* polymorphism may lead to low serum GNLY concentrations, these inherent genotypes were not associated with IGRA-positive status, indicating that low GNLY concentrations may be a consequence rather than a cause of LTBI.

Sputum smear-positive cases of TB have long been targeted in TB control programs [25] and preventive therapy for LTBI in the general population has not been feasible in most countries with high TB burdens, including in Vietnam. However, identification of subgroups of individuals who are at high risk of developing active disease may provide a useful and cost-effective strategy to control TB [26]. Thus, future investigations are required to explore the roles of GNLY and other antimicrobial molecules as biomarkers, for example, in prospective cohort studies with follow up of blood levels during the first two years after infection.

Our study has some limitations. We could not recruit individuals with no TB exposure. We compared our results with those obtained from previous studies and discussed possible interpretations of our findings. Because previous studies of granzymes [8] and perforin [7] reveal that GNLY works in cooperation with these molecules to eliminate MTB, it may be of further value to investigate the co-localization of these effector molecules using flow cytometry or cell population analyzers to offer greater certainty of this assertion. Moreover, the 15-kDa precursor form of GNLY is better known as a potent chemotactic factor during inflammation than as a bactericidal molecule. Since the present sandwich ELISA system captures both forms of GNLY, further studies are required to determine which form of GNLY is predominant in blood from patients with LTBI and active TB. Lastly, the SNP rs11127 (C/T) of exon 4 leads to an amino acid substitution (Ile104Thr) in the GNLY protein and may accelerate consumption or enhance stability of GNLY. Since *GNLY* genotype was associated with GNLY concentration, but not with mRNA expression level, we can assume that *GNLY* polymorphism may have affected GNLY concentrations at the protein level; and *GNLY* may have been expressed in the way partly independent of *GNLY* genotype. A genetic variation that directly affects GNLY concentrations was not clearly determined in this study because of strong linkage disequilibrium around the *GNLY* locus. Nevertheless, the SNP rs11127 has been reported to have an important role in clearance of hepatitis B virus [27, 28], and further study on GNLY genetic polymorphism is necessary whether it may contribute to optimize granulysin vaccines against TB [29, 30].

## Conclusion

Serum GNLY concentrations in IGRA-positive participants were significantly lower than in those with IGRA-negative status. Therefore, changes in circulating GNLY, presumably at the protein level, may be involved in the immunological condition of LTBI.

## Abbreviations

BCG: Bacillus Calmette-Guérin
CI: Confidence interval
CTL: Cytotoxic T lymphocytes
DOTS: Directly observed treatment short-course
ELISA: Enzyme-linked immunosorbent assay
GNLY: Granulysin
IGRA: Interferon-gamma release assays
LTBI: Latent tuberculosis infection
MTB: *Mycobacterium tuberculosis*
NK: Natural killer
OR: Odds ratio
PCR: Polymerase chain reaction
SNP: Single nucleotide polymorphism
TB: Tuberculosis
